# Influence of Immunology Knowledge on Healthcare and Healthy Lifestyle

**DOI:** 10.1371/journal.pone.0159767

**Published:** 2016-07-28

**Authors:** Noor Lide Abu Kassim, Afiqah Binti Saleh Huddin, Jamal Ibrahim Daoud, Mohammad Tariqur Rahman

**Affiliations:** 1 Faculty of Dentistry, International Islamic University Malaysia, Kuantan Campus, Pahang 25200, Malaysia; 2 Faculty of Nursing, Umm Al-Qura University, Mecca, Saudi Arabia; 3 Department of Biotechnology, Faculty of Science, International Islamic University Malaysia, Kuantan Campus, Pahang 25200, Malaysia; 4 Faculty of Engineering, International Islamic University Malaysia, Gombak, Kuala Lumpur 53200, Malaysia; 5 Faculty of Dentistry, University Malaya, Jalan Universiti, Kuala Lumpur 50603, Malaysia; University of Malaya, MALAYSIA

## Abstract

Completing a course in Immunology is expected to improve health care knowledge (HCK), which in turn is anticipated to influence a healthy lifestyle (HLS), controlled use of health care services (HCS) and an awareness of emerging health care concerns (HCC). This cross-sectional study was designed to determine whether these interrelationships are empirically supported. Participants involved in this study were government servants from two ministries in Malaysia (n = 356) and university students from a local university (n = 147). Participants were selected using the non-random purposive sampling method. Data were collected using a self-developed questionnaire, which had been validated in a pilot study involving similar subjects. The questionnaire items were analyzed using Rasch analysis, SPSS version 21 and AMOS version 22. Results have shown that participants who followed a course in Immunology (CoI) had a higher primary HCK (Mean = 0.69 logit, SD = 1.29 logits) compared with those who had not (Mean = -0.27logit, SD = 1.26 logits). Overall, there were significant correlations among the HLS, the awareness of emerging HCC, and the controlled use of HCS (*p* <0.001). However, no significant correlations were observed between primary HCK and the other variables. However, significant positive correlation was observed between primary HCK and controlled use of HCS for the group without CoI. Path analysis showed that the awareness of emerging HCC exerted a positive influence on controlled use of HCS (β = 0.156, *p* < .001) and on HLS (β = 0.224, *p* < .001). These findings suggest that having CoI helps increase primary HCK which influences controlled use of HCS but does not necessarily influence HLS. Hence, introducing Immunology at various levels of education and increasing the public awareness of emerging HCC might help to improve population health *en masse*. In addition, further investigations on the factors affecting HLS is required to provide a better understanding on the relationship between primary HCK and HLS.

## Introduction

Health literacy represents the necessary skills for an individual to appropriately and effectively act in the health care environment and use health related information. Health literacy has been defined as “the degree to which individuals have the capacity to obtain, process, and understand the basic health information and services they need in order to make appropriate health decisions” [1]. Hence, health literacy is recognized as a factor that affects one’s ability to utilise health information and services for favourable health outcomes [2].

Promotion of health literacy includes community-based education intervention towards specific health concerns; such as cardiovascular diseases, and vaccination against human papiloma virus (HPV) [3, 4, 5]. For example, educating mothers on the dangers of HPV encouraged them to have a more positive response towards HPV vaccination [5]. School-based health education programs intended to prevent teenage substance abuse was found to foster positive health behaviour choices among teenagers [6]. While evaluating “Child and Adolescent Trial for Cardiovascular Health”, Hoelscher and colleagues showed that prior training had the greatest impact on positive health behaviours while staff training was identified as an important factor in achieving institutionalization of school-based health education programs [3]. Together, in addition to comprehensive communications with physicians, health literacy or health education plays important roles in the (i) maintenance of a healthy lifestyle (HLS), (ii) awareness of emerging health care concerns (HCC) such as emerging diseases and their prevention, (iii) prudent use of health care services (HCS) such as vaccinations, antibiotics and over-the-counter (OTC) drugs.

Following a course in immunology (CoI) typically provides basic and applied knowledge in cellular and molecular biological mechanisms of the body related to the physiological state of health as well as protection against pathological conditions. Thus it is expected that an individual with formal institutional training in Immunology, (i.e., having CoI) would have higher outcomes in HLS, greater awareness for emerging HCC and would be more prudent in the use of HCS. Hence, this study aimed to evaluate whether having CoI enhances the primary health care knowledge (HCK) of an individual, and whether this (HCK) would in turn influence one’s HLS, the controlled use of HCS, as well as the awareness for emerging HCC.

## Materials and Methods

### Study design and instrumentation

In this study, a cross-sectional questionnaire (S1 File) survey design was used. The questionnaire was validated by experts for its content validity prior to a pilot study. A pilot study was conducted to further validate the internal consistency and validity of the questionnaire items. The questionnaire consisted of four main sections representing four constructs: HLS, HCS, HCK and HCC. Section A consisted of questions on HLS, which included sleep behaviour, regularity of exercise, dietary intake and cleanliness. Section B pertained to controlled use of HCS, such as, frequency of taking vitamin supplements, completion of prescribed doses of antibiotics, and dependence on OTC drugs for common ailments such as headache and common flu. Section C consisted of 13 questions, assessing primary HCK such as knowledge of: the uses of vaccines, the role of the body defense mechanism (i.e., the immune system), and the role of medicine in treating a disease. Section D included questions on awareness of HCC. The best answer to the items in sections A and B were given the highest score (5) whereas the least correct/appropriate answers were given lowest score (1). Correct answer to items in section C were assigned a score “1” while incorrect answers were assigned a score “0”.

The questionnaire was administered without any section subheadings and the questions were arranged in a general logical order to avoid biasness in participants’ responses. Internal consistency (Cronbach alpha) values of the measured variables are as follows: Emerging HCC (.83), primary HCK (.60), HLS (.56). Values for primary HCK and HLS were rather low due to some randomness in respondents’ responses to several items. Nonetheless, all items were included in the final analysis despite their low item-total correlation for reasons of content representativeness.

### Ethics approval

Prior to the distribution of the survey forms to the participants, the research was approved by the Medical Research and Ethics Committee, Malaysian Ministry of Health (Jawatankuasa Etika and Penyelidikan Perubatan, Kementerian Kesihatan Malaysia) [Ref: KKM/NIHSEC/08/0804/P12-512]. Additional written consent form was neither attached with the ethics approval application nor provided during the questionnaire distribution to the participants. This is because each questionnaire had the statement “Your participation is completely voluntary. Information given will be used solely for research and educational purposes. Personal information will be kept confidential in the dissemination of the research findings. If you do not wish to participate, please do not fill this form.” (S1 File) which was supplied to the Medical Research and Ethics Committee. Hence it was apparent that both the Medical Research and Ethics Committee and the participants would recognize that participation in the survey is voluntary and the data to be used solely for research purposes without disclosing any information or the identity of the participants. Notably, the questionnaire was self-administered (i.e., once distributed in person, participants were requested to complete the questionnaire by themselves).

### Participants

A total of 804 questionnaires were distributed during the period of March-August in 2012, to two groups of participants: (i) university students, studying Biotechnology and Biomedical science at the undergraduate level in a Malaysian university and (ii) government servants who are working at the Ministry of Education, Higher Education, and Health in Malaysia. Among them, 78 declined to participate in the study while the other 192 did not respond and 21 submitted incomplete questionnaires. Finally, 513 questionnaires were analyzed for the study, which constituted a response rate of 63.81%. Participants were further categorized depending on whether they had undergone any course on Immunology (CoI) during their tertiary education.

The participants were selected using non-random purposive sampling procedure. This procedure was used in order to compare between sub-groups with selected characteristics, namely formal instruction and knowledge of immunology. They were also selected to represent Malaysian young and older adults. The sample size was calculated based on 95% CI and 5% margin of error.

### Statistical analysis

The questions/items for each section measuring the variables of this study were analysed using Rasch analysis to produce individual person measure for each variable [7]. The unit of measurement used in Rasch analysis is log odd units or logit. Rasch analysis was used as an analysis procedure as it allows for summation of responses of items in a subscale and transformation of the ordinal data from the likert scale into interval data through log odd transformation. Person measures in log odd units (Logits) derived from the Rasch analysis were then used in the statistical analyses to determine mean differences, correlations and regression weights [8]. The statistical tests utilized in the study were independent sample t-test, Pearson *r* correlation, and path analysis using AMOS version 22. Statistical significance was determined at p≤.05.

## Results

### Demographic characteristics of Participants

Socio-demographic characteristics of the participants are presented in Table 1. The average age of the participants is 30.5 years; with 30% male and 70% female. The majority of the participants were Malay (92.6%) and government servants (71.3%). Among the participants, 68.6% had formal CoI during their tertiary education. Since the study involved voluntary participation, and part of the study population are university students, the sample distribution concentrating between 20–30 years is not unexpected (Table 1).

**Table 1:** Demographic Characteristics of Respondents.

Characteristics	Frequency (n)	Percent (%)
Gender		
Male	154	30.0
Female	359	70.0
Race		
Malay	475	92.6
Chinese	10	1.9
Indian	13	2.5
Others	14	2.8
Missing	1	0.2
Age group (years)		
20–30	332	64.7
31–40	87	17.0
41–50	66	12.8
51–60	28	5.5
Mean Age (30.5)		
Nationality		
Malaysian	507	98.8
Non-Malaysian	5	1.0
Missing	1	0.2
Course in Immunology (CoI)		
Yes	152	29.2
No	361	68.6
Total (N)	513	100.0

### Mean scores for primary HCK, HLS, Controlled use of HCS, and awareness for emerging HCC

As assumptions of normality and independence were satisfied, the mean scores of primary HCK, HLS, controlled use of HCS and awareness of emerging HCC were compared between groups with regards to having CoI using the independent sample t-test. The results showed that participants who received CoI scored significantly higher on primary HCK (*p* = .000) compared to those without. There were no significant differences between the two groups (*p*≤.05) for HLS, controlled use of HCS, and awareness for emerging HCC (Table 2).

**Table 2:** Independent sample t-test results and comparison of mean scores between Groups for primary HCK, HLS, controlled use of HCS and awareness of emerging HCC.

Groups	n	Mean (SD) logits	t	*p*-value
*Primary HCK*				
Respondents without CoI	361	-0.27 (1.26)	-7.884	.000**
Respondents with CoI	152	0.69 (1.29)		
*Healthy Lifestyle*				
Respondents without CoI	361	0.52 (0.30)	-0.677	.499
Respondents with CoI	152	0.54 (0.31)		
*Controlled Use of HCS*				
Respondents without CoI	361	0.23 (0.27)	1.125	.261
Respondents with CoI	152	0.20 (0.29)		
*Awareness for emerging HCC*				
Respondents without CoI	361	0.61 (2.69)	-1.550	.122
Respondents with CoI	152	1.03 (2.91)		

** Correlation is significant at the .01 level (2-tailed).

### Primary HCK in using vaccine and medicine is significantly different among the participants with and without CoI

Since we found a significant difference in primary HCK between the groups with and without CoI (Table 2), we further evaluated the areas in which formal instruction or course in Immunology had an impact on primary HCK. It was found that significantly higher number of participants with CoI (+CoI) gave correct answers to questions related to function and use of vaccines and antibiotics (Table 3).

**Table 3:** Distribution of participants with correct answer to questions on primary HCK.

Domain of primary HCK	No.	Questions to evaluate primary HCK	% respondents		p-value
			+ CoI	- CoI	
Etiology of disease	1	It is important to know the donor’s history of diabetes and blood pressure for blood transfusion	20.4	16.1	0.237
	2	Mental and physical stress can cause heart diseases	89.5	88.9	0.918
	3	Mental and emotional stress can increase the risk of physical illness and infectious diseases	68.4	60.1	0.076
	4	Both bacteria and virus are living organisms	38.8	19.1	0.000**
	5	When a person is allergic to some food, it means that the person’s digestive system is not functioning properly	90.1	64.0	0.000**
Use of medicine	6	Taking medicine to fight disease is necessary because our immune system can’t function efficiently without medicine	66.4	48.8	0.000**
	7	Antibiotic resistance of bacteria may be developed because of the weakening of the immune strength of an individual.	30.9	21.1	0.017*
	8	Finding an effective drug to treat AIDS is difficult because HIV attacks cells that are normally responsible to fight diseases	88.8	81.7	0.046*
	9	It is necessary to complete the prescribed dose of medicine once a physician prescribes it to us.	53.9	55.4	0.762
Use and role of vaccines	10	Vaccines are mainly composed of a chemical agent that stops bacterial or viral growth in the human body	57.2	24.9	0.000**
	11	Vaccines can prevent entry of disease causing virus or bacteria into our body	53.9	26.9	0.000**
	12	Vaccines are used to treat a disease	63.2	49.9	0.007**
	13	Vaccination against influenza guarantees immunity against flu.	51.3	34.6	0.000**

* Correlation is significant at p < .05.

** Correlation is significant at p < .01.

**Fig 1:**
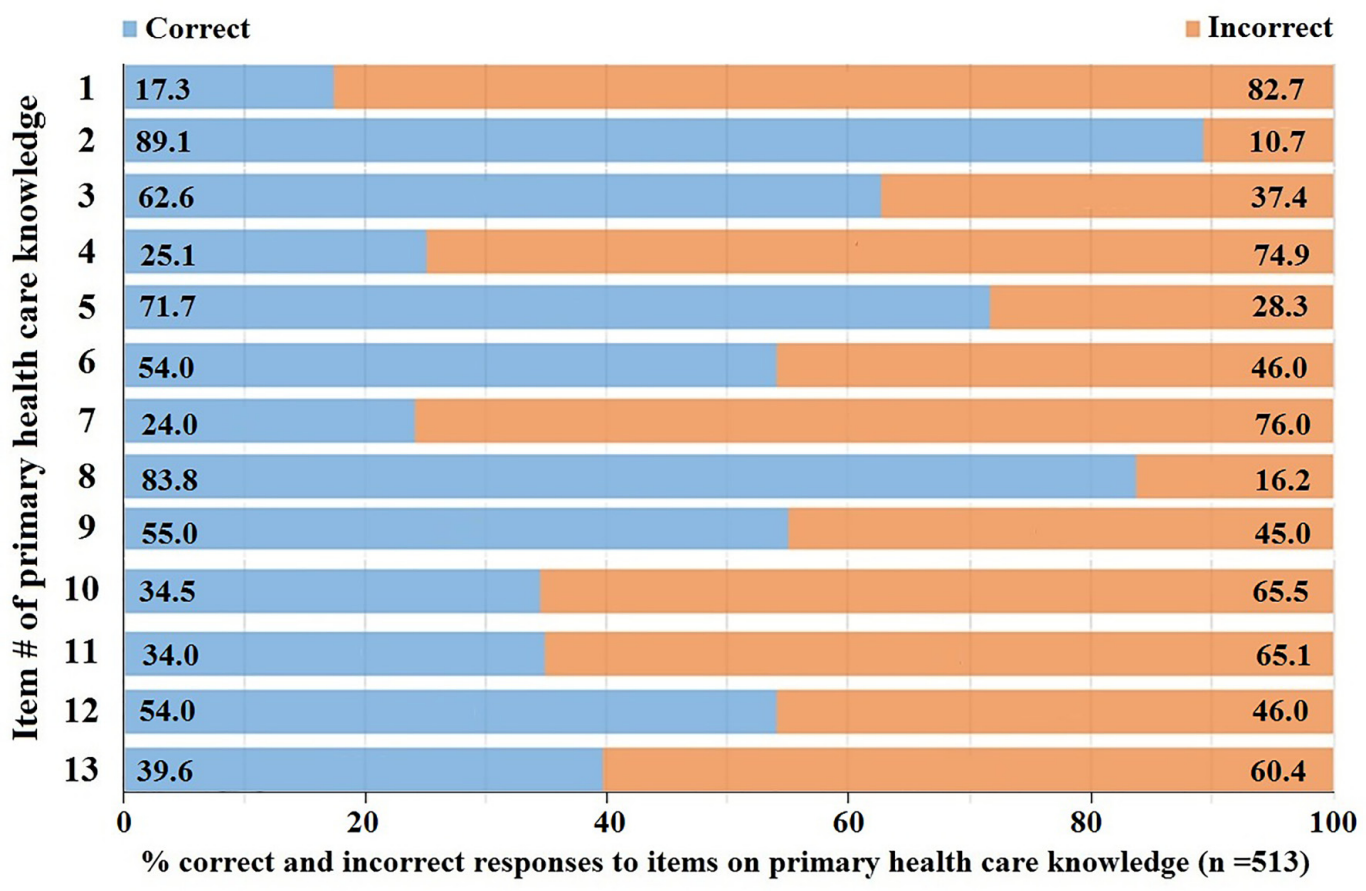
Distribution (%) of correct and incorrect responses to the items evaluating primary HCK. Irrespective of having CoI during tertiary education, only few items (item # 2,5, and 8; items are described in Table 3) were answered correctly by the majority (>50%) of the participants.

When the percent distribution of the correct and incorrect responses to the items among all the participants were evaluated, irrespective of exposure to CoI, only few items (such as item # 2: Mental and physical stress can cause heart diseases, Item# 5: When a person is allergic to some food, it means that the person’s digestive system is not functioning properly, and item # 8: Finding an effective drug to treat AIDS is difficult because HIV attacks cells that are normally responsible to fight diseases) were answered correctly by the majority of the paritcipants (Fig 1). All other items had low percent correct responses.

### Association among primary HCK, HLS, controlled use of HCS, and awareness of emerging HCC

Relationships among primary HCK, HLS, controlled use of HCS, and awareness of emerging HCC were estimated using Pearson *r* correlation coefficient. In the overall group analysis, significant positive correlations were observed among the following variables: awareness of emerging HCC, HLS, and controlled use of HCS (*p* < .01) (Table 4). The same analysis was performed to examine the correlations among the variables for each participant group (with and without CoI). Similar results were found for the group with CoI. However, for the group without CoI, showed a significant positive correlation which was observed between primary HCK and controlled use of HCS (Table 4).

**Table 4:** Association among primary HCK, HLS, controlled use of HCS, and awareness for emerging HCC.

Correlation between	*r*	*p-*value
**All respondents (n = 513)**		
Primary HCK and HLS	-.019	.670
Primary HCK and controlled use of HCS	.078	.077
Primary HCK and awareness for emerging HCC	.075	.088
HLS and controlled use of HCS	.220	.000**
HLS and awareness for emerging HCC	.221	.000**
Awareness for emerging HCC and controlled use of HCS	.202	.000**
***Group*: *Without CoI (n = 361)***		
Primary HCK and HLS	-.005	.919
Primary HCK and controlled use of HCS	.109	.038*
Primary HCK and awareness for emerging HCC	.074	.162
HLS and controlled use of HCS	.204	.000**
HLS and awareness for emerging HCC	.195	.000**
Awareness for emerging HCC and controlled use of HCS	.189	.000**
***Group*: *With CoI (n = 152)***		
Primary HCK and HLS	-.087	.289
Primary HCK and controlled use of HCS	.080	.325
Primary HCK and awareness for emerging HCC	.018	.824
HLS and controlled use of HCS	.262	.001**
HLS and awareness for emerging HCC	.273	.001**
Awareness for emerging HCC and controlled use of HCS	.242	.003**

* Correlation is significant at the .05 level (2-tailed).

**Correlation is significant at the.01 level (2 tailed).

### Controlled use of HCS is influenced by HLS, and awareness of emerging HCC

**Fig 2:**
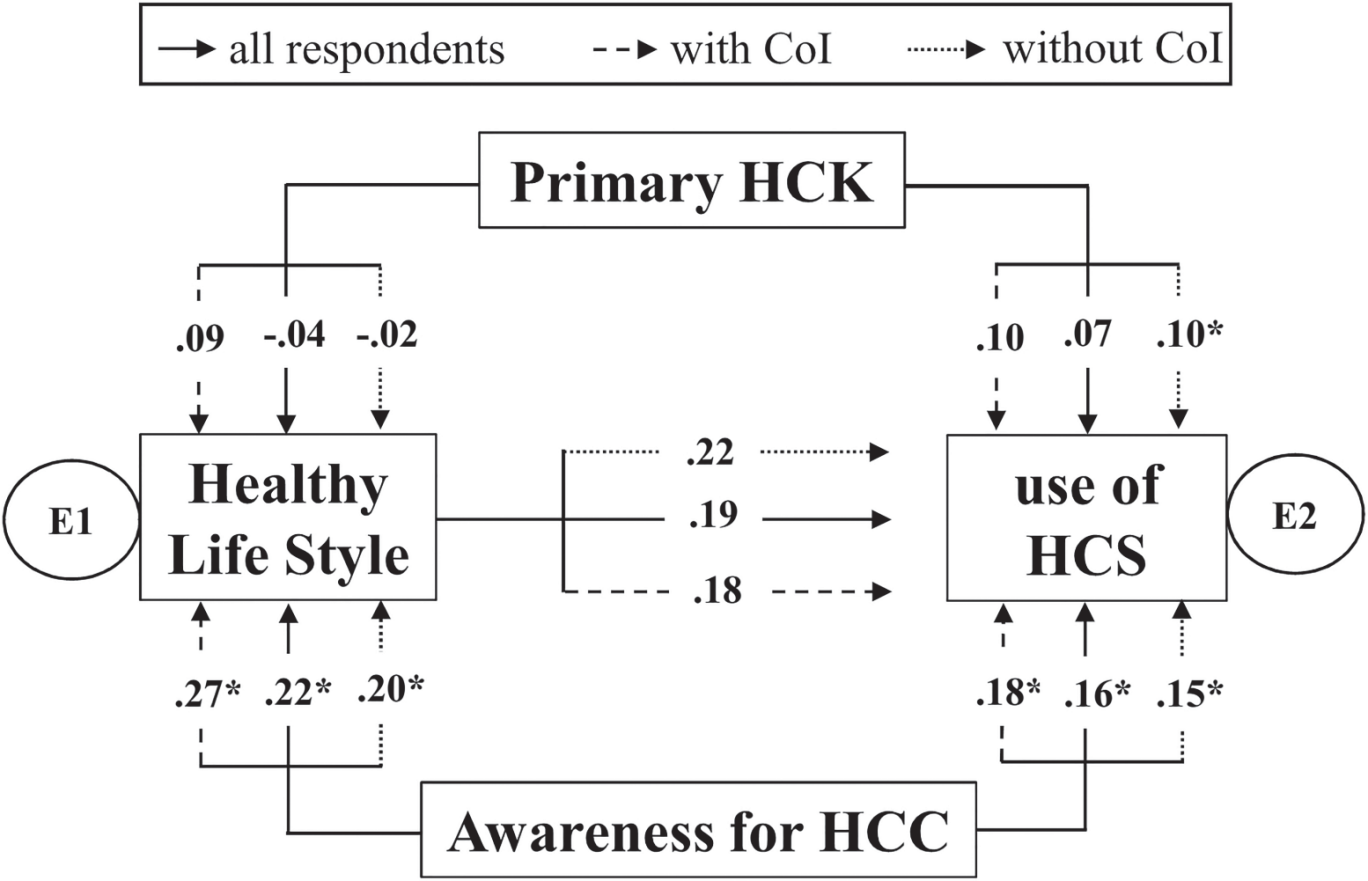
Interrelationships among primary HCK, HLS, awareness of emerging HCC and controlled use of HCS. Awareness of emerging HCC showed significant associations (marked as *) with healthy lifestyle practice and controlled use of HCS (*p* < .001) irrespective of background in Immunology (i.e., having CoI) during the tertiary education. At the same time, HLS showed significant influence on the controlled use of HCS (*p* < .001). The association between HCK and controlled use of HCS for participants without CoI was significant at p = .050. E1 and E2 refer to sets of unspecified cases of the effect variables (i.e., HLS and use of HCS). They are analogous to the error or residual in a predictive equation in multiple regression.

The interrelationships among primary HCK, HLS, controlled use of HCS, and awareness of emerging HCC were further investigated at the multivariate level using path analysis. In this analysis, primary HCK was hypothesized to influence HLS and controlled use of HCS. It was also hypothesized that awareness of emerging HCC influenced HLS and controlled use of HCS. Healthy lifestyle was postulated to mediate the influence of awareness of emerging HCC on controlled use of HCS (Fig 2).

The goodness-of-fit indices showed a good model-data fit [χ^2^ (1) = 2.912; RMSEA = .061; CFI = .971; *p* = .088]. The all group analysis indicated a statistically significant influence of awareness of emerging HCC on HLS (standardized regression weight β = 0.224; *p* < .001) and on controlled use of HCS (standardized regression weight β = 0.156; *p* < .001). The influence of HLS on controlled use of HCS was also statistically significant (standardized regression weight β = 0.188; *p* < .001). The relationship between primary HCK and HLS and controlled use of HCS, on the other hand, were not statistically significant (*p*>.05). (Fig 2)

### Participants without CoI shows significant influence of primary HCK on controlled use of HCS

Multiple group analysis was also carried out to investigate the influence of having CoI on the interrelationships among the other variables. Goodness-of-fit indices showed good model-data fit (χ^2^ (2) = 2.007; CMIN/DF = 1.003; RMSEA = .061; CFI = .971; *p* = .088). The results indicated a significant influence of primary HCK on controlled use of HCS for the group without CoI (standardized regression weight (β = .100; *p* = .05) as compared to those with CoI (standardized regression weight β = .096; *p* = .212). Influence of awareness of emerging HCS on HLS and controlled use of HCS was higher for those with CoI (Fig 2).

### Influence of having CoI on the preferred mode of treatment

**Fig 3:**
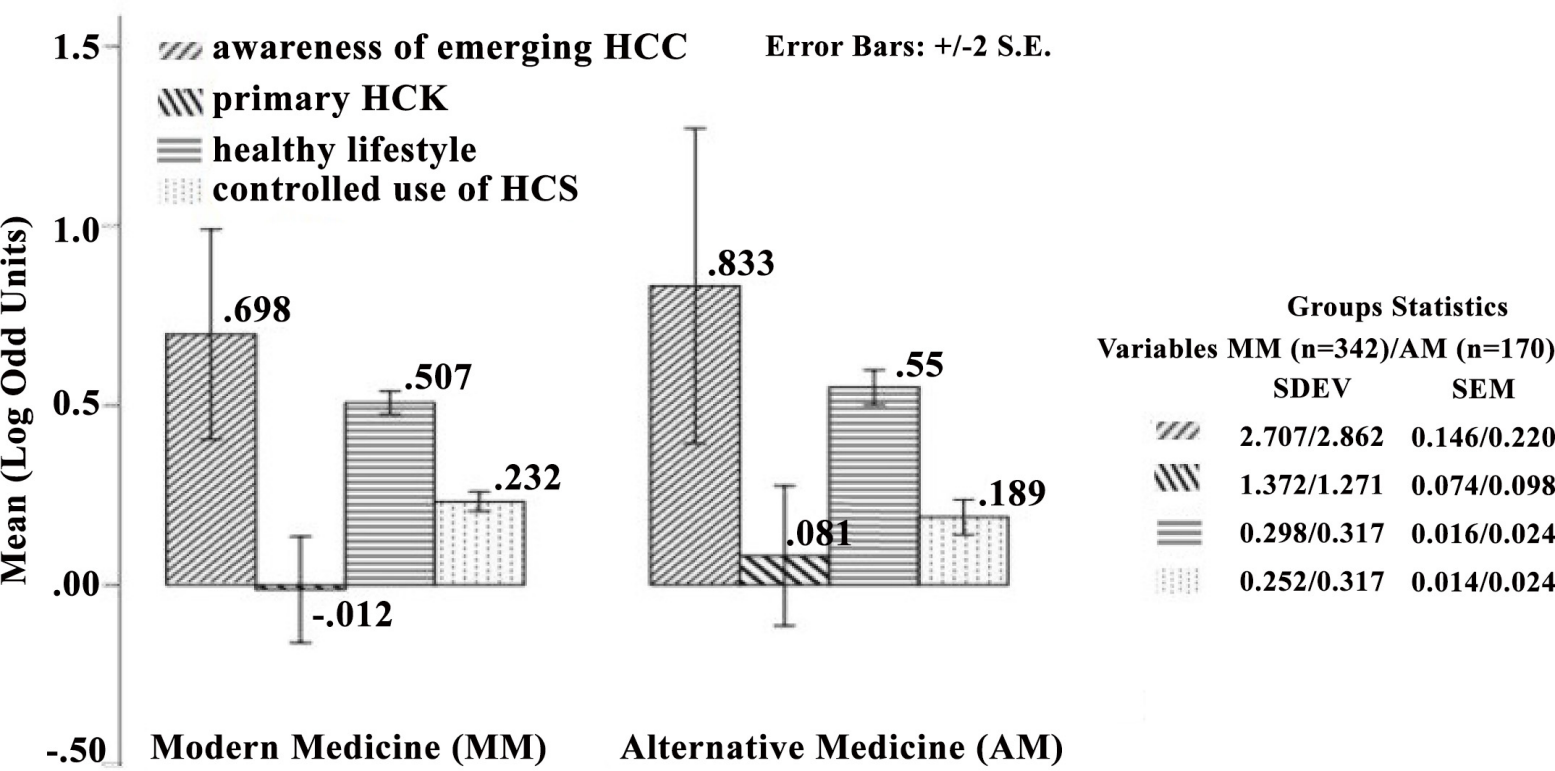
Influence of emerging HCC, primary HCK, HLS, and controlled use of HCS on the choice of modern medicine and alternative forms of medicine. No significant differences were found across the following variables: awareness of emerging HCC, primary HCK, HLS, and controlled use of HCS among those who preferred either modern medicine (MM) or alternative medicine (AM).

Chi-square test of association showed no significant association between having CoI and the preferred mode of treatment (χ^2^ (3) = 1.595; *p* = .661), while independent sample t-tests showed no significant differences between those who preferred modern medicine and alternative medicine, across the following variables: awareness of emerging HCC, primary HCK, healthy lifestyle, and controlled use of HCS (Fig 3).

## Discussion

Education plays a key role in promoting a healthy lifestyle as well as in preventing diseases [9]. However, health education or health literacy programs were found to be effective only among the most educated and economically privileged population [10]. For example, among South Korean men, individual level of education was found to be correlated with healthy lifestyle parameters such as regular physical exercise and health check-up [11]. The field of health literacy has made tremendous advancement with significant impact on health outcomes [2]. Mostly, health literacy programs have attempted to address specific target groups; such as to promote maternal and child health, to prevent communicable disease, and to promote immunization and other preventive health services [11]. For example, a community-based education intervention (a 60 min power-point presentation, understanding HPV including group discussion) to increase HPV related knowledge of mothers of vaccine eligible daughters have been shown to significantly increase mothers’ intent to vaccinate their daughters [5]. Other specific target group health literacy intervention of this nature include: risk groups for CVD and HIV risk groups [4,12].

Although this strategy has been found to be effective, novel approaches to increase motivation; improved techniques for delivering written, oral, or numerical information; and “work-around” interventions such as patient advocates have been recommended to improve health literacy outcomes [2]. Therefore, more conceptual and empirical research are required to understand the impact of health literacy on the use and outcomes of health care. Empowering the future generation with at least fundamental knowledge of Immunology could provide a component of holistic approach of health literacy and eventually the overall health outcomes [13]. Therefore, this study attempted to evaluate how following a course in Immunology at tertiary level of education would affect one’s primary (basic) level of health care related knowledge and its influence on one’s healthy lifestyle, controlled use of health care facilities, and awareness of emerging health care concerns.

The initial challenge for the study was to identify suitable items to evaluate primary HCK of an individual. The items are expected to cover important aspects of health care concerns and should not be technically unknown to the general public. Health care by definition is commonly known as “the prevention, treatment, and management of illness and the preservation of mental and physical well-being through the services offered by the medical and allied health professions”. Therefore health care includes three important aspects of disease control: prevention, treatment and cure. One’s knowledge on the etiology i.e., cause of disease as well as means to prevent or avoid spread of the disease causing agents is important for the prevention of disease. Thus awareness of the etiology and use of vaccine against an infectious agent are important aspects of health care concerns. Once being in the diseased state, treatment becomes the most important aspect of health care that requires following of the instructions given by physicians, such as proper utilization of prescribed medicines. Therefore, questions from three aspects of health care concerns namely: (i) general etiology of disease, (ii) knowledge of use of medicines, and (iii) knowledge of vaccine use were chosen to evaluate the primary HCK (Table 3).

Identifying the questions to evaluate controlled use of health care services was also critical since no reported study was found in the area. Formulating items for the survey questionnaire for this section was based on the principle that an individual should not use medicine without prescription, which is a common practice in many nations such as Belgium [14], Croatia [15], Jordan [16] and Malaysia [17]. On the other hand, once advised by physicians, the prescribed use of medicine given for the treatment should be followed. For example, a failure to complete a prescribed course of antibiotics can cause immediately unseen health complications such as antibiotic resistance [18]. Items chosen to evaluate the awareness of emerging health care concerns was based on how often an individual makes efforts to learn about the emerging health related issues from health related magazines, newspapers or other forms of media.

Choosing items to evaluate healthy life style was mostly based on published surveys on life style studies [19, 20, 21]. Items related to health dietary habits and physical activity (exercise) were taken into consideration and included for the current survey. However, some common aspects of healthy life style that were used by other researchers were not included for the final evaluation of the current study because of the demographic profile of the majority of the participants. For example, questions related to smoking and alcohol consumption, although initially included, were finally not considered as majority of the participants (~93%) were Malay Muslims (Table 1).

It is interesting to note that the majority of the participants, irrespective of their exposure to CoI, could respond correctly only to a few items regarding primary HCK (Fig 1). These items are related to whether mental and physical stress are linked to infectious diseases, why finding treatments for HIV is difficult, and nature of complications in food allergy. Notably, in recent years, stress related illness has been a major focus, especially in developed countries. And mass media plays an important role to promote awareness for mental and physical illness. Similarly, focus on HIV education in all media has also been observed for the last few decades, while food allergy is a common phenomenon in many Malaysian families [22]. Thus, people in general are more aware of these issues. Hence it was not unexpected that majority of the participants, irrespective of their exposure to CoI, could respond correctly to the related items.

As expected, the respondents with CoI scored significantly higher on primary HCK (p≤.05) compared to those without CoI. However, the level of primary HCK did not influence their HLS, controlled use of HCS, or their awareness of emerging HCC (Table 2). In the overall group (all respondents with and without CoI) analysis, awareness of emerging HCS was found to be significantly correlated with HLS, and controlled use of HCS (p≤.05). Ironically, for the group without CoI, a significant positive correlation was observed between primary HCK and controlled use of HCS (Table 3). Their HLS also showed a significant positive correlation with controlled use of HCS (p≤.05) (Fig 1). Similar correlations were found for the group with CoI (Table 3). Besides, it was expected that practice of self-medication that is part of controlled use of HCS, such as taking over the counter drugs, could be influenced by CoI or level of primary HCK. However, it can be assumed that since all the participants had either completed or were currently pursuing their tertiary education therefore, their level of education might have influenced the controlled use of HCS. Consistently, it was reported that poor knowledge of self-medication can lead to common practice of self-medication which in most cases is inappropriate [23]. Length of education has also been shown to have an effect on healthy dietary habit [22]. Healthy diet is an important aspect of HLS.

In addressing antibiotic resistance, Lee and colleagues reviewed the importance of minimal use of antibiotics and prudent antibiotic prescription by physicians [24]. Efforts to educate medical professionals were found significantly effective in reducing antibiotic prescribing. The World Health Organization (WHO) has emphasized the importance of the public within the community as well as professionals in the control of antibiotic resistance [25]. For example, poor knowledge of asthma was found to be strongly correlated with improper metered-dose inhaler use among asthma patients [26]. Thus educational programs to promote proper use of antibiotics by patients are also expected.

However health care knowledge cannot be the sole factor influencing different aspects of health care including healthy lifestyle, awareness for emerging health care concerns and controlled use of health care services. This is consistent with the notion that knowledge alone does not guarantee the behavioural modification, especially for younger generations [27].

This study also looked at whether awareness of health care concerns or controlled use of health care services is influenced by preference for therapeutic interventions such as modern medicine or any other forms of complementary or alternative medicines (such as Chinese medicine, Homeopathy or Ayurveda). It was found that the influence of the primary HCK, awareness of HCC or controlled use of HCS on the preference of therapeutic interventions were nonsignificant (Fig 3). The existence and use of different health care alternatives in Malaysia with three major ethnic populations (Malay, Chinese and Indian) might have influenced the popularity of all forms of alternative medicine in addition to Modern medicine. Besides, the increasing use of complementary and alternative forms of medicine have been reported in 15 countries including Australia, Germany Japan, Malaysia, Singapore, and the United States of America [28].

It is expected that the current study which was based on participants from Malaysia will encourage similar studies in different parts of the world. It is not unlikely that further studies might have different outcomes depending on the academic, demographic as well as socio-economic background of the participants. Since majority of the participants in the study were adults in the age group of 20–30, the findings would be more representative for this age group and may not adequately reflect older age groups. Influence of depth of health care knowledge should also be evaluated to aid health care policy makers to adopt appropriate strategies of educating the population for health care, healthy life style as well as the use of health care services.

## Supporting Information

S1 File Questionnaire used to conduct the survey. (PDF)
