# Supplementary material for: Influence of Immunology Knowledge on Healthcare and Healthy Lifestyle
Source: PLoS One. 2016 Jul 28;11(7):e0159767. doi: 10.1371/journal.pone.0159767 (PMC4965037; doi:10.1371/journal.pone.0159767)
Supplement: S1 File — (PDF) [file pone.0159767.s001.pdf]

Supporting Information

S1 File: Questionnaire used to conduct the survey

SURVEY ID: _______________
FOR RESEARCHER USE ONLY

SURVEY

Your participation is completely voluntary. Information given will be used solely for research and educational purposes. Personal information will be kept confidential in the dissemination of the research findings. If you do not wish to participate please do not fill this form.

EXCLUSION CRITERIA:

1. Suffering from any form of mental illness.
2. Suffering from any form of physical disabilities.
3. Suffering from any chronic illness that require regular prescriptions
4. No formal education.
5. Age below 25 years and above 50 years.

PERSONAL INFORMATION

Age on January, 2011 : _____________
Sex : _____________

ACADEMIC BACKGROUND

Please mark (√) the highest level of education of your parent/guardian, spouse and yourself:

	Primary	Lower Secondary	Upper Secondary	Diploma	Tertiary
SELF					
SPOUSE					
GUARDIAN/PARENT					

Do you have any formal training/education in Immunology? (Please tick √)

**Yes**		**No**	

1. Health has different dimensions as mentioned below. Rate these dimensions in your opinion in order of importance.

[Key: 5= Most Important; 1= Least Important].

	1	2	3	4	5
**A.** Physical Health: ability of the body, organs, tissues to function/work properly					
**B.** Mental health: ability to think clearly and coherently					
**C.** Emotional health: ability to recognize and appropriate expression of fears, joys, grief, anger.					
**D.** Social health: ability to make and maintain relationship with other in the society					
**E.** Spiritual health: ability to uphold religious belief and practice according to religious teachings					

2. Rate the following activities in terms of its contribution towards a healthy nation.

**A.** Celebrating a day of healthy living every year to promote awareness					
**B.** Promoting healthy life style through TV/Radio/other media					
**C.** Providing free healthcare services such as free medical check-ups					
**D.** Providing health classes in schools as well as for the public					
**E.** Supporting the health sector by providing more funds for R&D and the use of cutting-edge healthcare technology					
**F.** It is important for children to learn about the body's defence mechanisms (immunology) in school.					

3. What kind of treatment option do you prefer?

A. Modern Medicine B. Homeopathy C. Traditional/Herbal/Ayurvedic D. Other: __________

4. Why do you prefer that particular mode of treatment?

Rate (√) the following statements. [Key: 1 = Strongly disagree; 5 = Strongly agree]

	1	2	3	4	5
**A.** Social taboo for other options					
**B.** Most effective as I know					
**C.** Financial reason					
**D.** Availability					

Section A: Healthy life style

1. **How long do you sleep on average per day?**

A.< 6 hrs　B.6-8 hrs　C.> 8-10 hrs　D.>10 hrs　E. Irregular

2. **How many days a week do you have physical activity/exercise (equivalent to at least 30 min of walking per day)?**

A. Everyday　B. 5-6 days　C. 3-4 days　D. 1-2 days　E. Irregular

Specify your DAILY average consumption of the following (one glass = 250 mL).

**3. Water**	A. ≤6 glasses	B. 4-5 glasses	C. 2-3 glasses	D. 1 glass	E. < 1 glass
**4. Milk**	A. > 2 glasses	B. 1-2 glasses	C. < 1 glass	D. Irregular	E. Never
**5. Tea/Coffee**	A. > 4 cups	B. 2-3 cups	C. < 2 cups	D. Occasionally	E. Never
**6. Soft drinks**	A. > 250mL	B. 100-250mL	C. < 100mL	D. Irregular	E. Rarely
**7. Meat/Fish**	A. > 75g	B. 50-75g	C. < 50g	D. Rarely	E. Never
**8. Vegetables/Fruits**	A. > 100g	B. 50-100g	C. < 50g	D. Rarely	E. Never

**Rate (√) the following statements. [Key: 1 = Rarely; 5 = Regularly]**	1	2	3	4	5
**9. I brush my teeth**					
Every morning after waking up					
Before I sleep at night					
After every meal					
**10. When I come in from outside**					
In the morning after waking up					
Before going to bed					
Before handling food					

Section B: Use of Health care services

1. Will you take those vaccines which are NOT obligatory according to the Government health care policies?

Yes
Why?
No.
Why not?
Others:

2. How often do you take vitamins, minerals or other supplements although you have no weakness or disease?

A. Only upon doctor's advice　B. Daily　C. Weekly　D. Rarely　E. Irregularly

3. How often do you complete the prescribed course of antibiotics?

A. Always　B. Often　C. Sometimes　D. Occasionally　E. Never

**Rate (√) the following statements. [Key: 1 = Rarely; 5 = Frequently]**	1	2	3	4	5
**4. When suffering from common ailments (such as headache, fever or indigestion)**					
I take OTC (over-the-counter) drugs immediately					
I seek medical advice if symptoms last >24 hours					
I take OTC drugs if symptoms last more than 24 hours					
I seek medical advice if I am unable to engage in daily activities					
I seek medical advice immediately					
**5. I take medicine with doctor's advice**					
**6. I take a lot of water/saline**					
**7. I continue eating as usual**					
**8. I take OTC drugs**					
**9. I skip two or three heavy meals**					

Section C: Primary Health care Knowledge

For the following questions, circle TRUE for a correct statement, or FALSE for an incorrect statement.

**1.**	It is important to know the donor's history of diabetes and blood pressure to use his blood for blood transfusion	**TRUE**	**FALSE**
**2.**	Mental and physical stress can cause diseases of the heart	**TRUE**	**FALSE**
**3.**	Mental and emotional stress can increase the risk of physical illness and infectious diseases.	**TRUE**	**FALSE**
**4.**	Both bacteria and virus are living organisms	**TRUE**	**FALSE**
**5.**	Taking medicine to fight disease is necessary because our immune system can't function efficiently without medicine	**TRUE**	**FALSE**
**6.**	Vaccines are mainly composed of a chemical agent that stops bacterial or viral growth in the human body	**TRUE**	**FALSE**
**7.**	Vaccines can prevent entry of disease causing virus or bacteria into our body	**TRUE**	**FALSE**
**8.**	Vaccines are used to treat a disease	**TRUE**	**FALSE**
**9.**	Vaccination against influenza guarantees immunity against flu.	**TRUE**	**FALSE**
**10.**	When a person is allergic to some food, it means that the person's digestive system is not functioning properly	**TRUE**	**FALSE**
**11.**	Antibiotic resistance of bacteria may be developed because of the weakening of the immune strength of an individual.	**TRUE**	**FALSE**
**12.**	Finding an effective drug to treat AIDS is difficult because HIV attacks cells that are normally responsible to fight diseases.	**TRUE**	**FALSE**
**13.**	It is necessary to complete the prescribed dose of medicine once a physician prescribes it to us.	**TRUE**	**FALSE**

Section D: Awareness for emerging health concerns

**Rate (√) the following statements. [Key: 1 = Rarely; 5 = Frequently]**	1	2	3	4	5
**1.** I read health-related articles online or in magazines/newspapers/books.					
**2.** I watch health-related programmes on television.					
**3.** I try to learn about as many diseases as I can					

In the event of a sudden epidemic or major public health concern such as H1N1 and Dengue

**4.** I make an effort to learn about it only if I am at risk					
**5.** I depend on media reports to learn about it					
